# Alterations in postmenopausal plasmatic lipidome

**DOI:** 10.1371/journal.pone.0203027

**Published:** 2018-09-04

**Authors:** Iara Antonia Lustosa Nogueira, Érika Joseth Sousa Nogueira da Cruz, Andréa Martins Melo Fontenele, José Albuquerque de Figueiredo Neto

**Affiliations:** 1 Postgraduate Program in Health Sciences, Federal University of Maranhão, São Luís/MA, Brazil; 2 University Hospital, Federal University of Maranhão, São Luís/MA, Brazil; 3 Postgraduate Program in Adult and Child Health, Federal University of Maranhão, São Luís/MA, Brazil; 4 Department of Pharmacy, Federal University of Maranhão, São Luís/MA, Brazil; 5 Department of Medicine I, Federal University of Maranhão, São Luís/MA, Brazil; Instituto de Investigacion Sanitaria INCLIVA, SPAIN

## Abstract

**Background:**

Menopause consists of a physiological process in women between 40 and 50 years of age, and it has substantial consequences for health, ranging from disturbances in lipid and glycidic metabolism to psychological stress and sleep alterations, thereby increasing women’s risk of cardiovascular diseases. Here, we attempted to identify potential lipid alterations not identified by the classic methods.

**Methods and results:**

We analyzed the serum lipid profile in 40 women in pre- and post-menopause using a lipidomic approach and mass spectrometry. Lipid species presented increased concentrations, with a difference of more than 25% post-menopause and with the ceramides (N.C23:0.Cer, N.C23:0(OH).Cer and N.C24:0(OH).Cer) standing out with a fold change of 1.68, 1.59, and 1.58, respectively. It was also observed that 14 metabolites presented a significant difference in the average concentrations between pre- and post-menopause, especially the ceramide species. Strong and positive correlations were identified between various metabolites and fasting glucose, glycated hemoglobin, total cholesterol, LDL, and triglycerides. Of note were the association ceramide (N.C10:0.Cer) and lysophosphatidylethanolamine (LPE.a.C18:0) between fasting glucose and glycated hemoglobin.

**Conclusion:**

This study detected lipid alterations, especially in ceramides, post-menopause, as well as correlations with glycidic and lipid markers, which may in the future be useful to investigate diseases associated with menopause.

## Introduction

Menopause consists of a physiological process in women between 40 and 50 years of age, signaling the end of reproductive ability. Menopause is associated with different signs and symptoms, ranging from disturbances in lipid and glycidic metabolism to psychological stress and sleep alterations. Moreover, this phase influences various risk factors for cardiovascular disease (CVD) [1, 2].

Women ranging from 50 to 65 years old present a considerable increase in cardiovascular risk, in that acute myocardial infarction and cerebrovascular accident represent 53% of the causes of death in this age group, with these being the main cause of mortality and surpassing breast cancer [3].

Millions of women live through the climacteric period, and there is a high prevalence of coronary arterial disease (CAD) among this group; therefore, understanding more about the lipid, hormonal, and metabolic alterations that occur in this period is of fundamental importance [3].

The classic analysis methods, in turn, present limitations, and in light of the number of people with metabolic diseases, there is a need for more detailed lipid analyses. It is therefore imperative that new tools and methodologies be sought both for diagnostic purposes and for monitoring the prescribed therapeutic efficiency, in order to enable the most adequate control and treatment of the disease and consequently preserve life [4].

Lipidomic integrated with other “omic” approaches, including metabolomic, proteomic and genomic, it is able to identify and quantify several metabolites of endogenous molecules within a biological system and has been successfully used to identifying metabolic profiles associated with biological activities, physiological state, and diseases [2, 5].

Studies that are relevant to the topic have already been published, such as Alshehry et al.[6], who found that sphingolipids, phospholipids, cholesterol esters and glycerolipids were associated with future cardiovascular events and cardiovascular death in people with type 2 diabetes mellitus; Zhang et al.[7], identified various metabolomic biomarkers in patients with ovarian epithelial cancer; and De Oliveira et al.[8], identified some lipids in greater abundance in the plasma of pregnant women in pre-eclampsia.

Ke et al.[2] identified metabolites as potential markers for menopause in Chinese women. Meanwhile, Havulinna et al.[9] discovered that different serum ceramides are associated with higher risk of adverse cardiovascular events in apparently healthy individuals.

Research on this topic in this stratum of the population is still emerging, so this study intends to fill part of this gap in knowledge.

The aim of this study was to assess the lipid profile in detail in order to identify potential changes, thus opening up a new perspective for understanding the metabolic disorders that post-menopausal women experience.

In this study, we determined the lipid profile of serum in 40 pre- and post- menopausal women using a lipidomic approach involving mass spectrometry.

## Material and methods

This cross-sectional study is part of the project called “Endothelial dysfunction and cardiovascular risk in climacteric women” carried out in the Gynaecology Service of the University Hospital of the Federal University of Maranhão (HUUFMA), in the municipality of São Luis, Maranhão state, in the northeast region of Brazil.

All women participated voluntarily. A free and informed consent form was obtained from each one of them after providing a complete explanation regarding their participation in the study. This research was approved by the Committee for Ethics in Research of University Hospital of Federal University of Maranhão under assessment n. 15/12 and 88/12.

### Study population

Women aged between 40 and 65 were chosen in the period from May to December of 2013.

The sample was divided into 2 groups: one “pre-menopause” group (n = 20), with women claiming to have a normal menstrual cycle, and another “post-menopause” group (n = 20), where the women had been amenorrheic for more than one year.

### Inclusion and non-inclusion criteria

Women between 40 and 65 years old and who took part in the first two stages of the research (interview and blood test) were included.

Women that presented the following conditions were not included: pregnancy, use of statins or hormone replacement therapy, those who had undergone coronary angioplasty or myocardial revascularization, those with a history of acute myocardial infarction, those who had no information on the cause of menopause and age that it occurred, or who entered into menopause due to surgical intervention, radiotherapy, or chemotherapy.

### Laboratory procedures

After fasting for 12 hours, 20mL of total blood was taken;12 mL of the blood was added to vacuum tubes with a gel and clot activator, and 8 mL was added to tubes with fluoride and sodium oxalate.

The blood samples were centrifuged for 10 minutes at 3000 rpm, and the supernatant was extracted. Three tubes with human serum were immediately sent for laboratory analysis, and 3 other tubes with 1.0 mL of serum were frozen at -80°C until analysis.

### Biochemical analyses

The laboratory tests (fasting glucose, total cholesterol (TC), high-density lipoprotein (HDL), triglycerides (TG), glycated hemoglobin, and high sensitivity C-reactive protein (hs-CRP), follicle-stimulating hormone (FSH), luteinizing hormone (LH), estradiol, progesterone and insulin)were conducted as recommended by the manufacturer using a COBAS 6000 automated analyzer (Roche Diagnostics®, Mannheim–Germany). The concentrations of low-density (LDL) and very low-density lipoproteins (VLDL) were calculated using the Friedwald formula (VLDL = TG÷5 and LDL = CT–HDL–VLDL)for triglyceride values of up to 400mg/dL [10].

### Lipidomic analysis

A targeted metabolomics approach was applied using Lipid Assay (it was analyzed species of Glycerophospholipids (162), Sphingolipids (33) and Ceramides (136)) by *Biocrates Life Sciences AG* (Innsbruck, Austria), according to internal standard protocol. The lipid classes were quantitatively analyzed in 40 human serum samples.

To determine the lipid metabolites, the samples (serum) were centrifuged, and the supernatant was used for analysis. Sample preparation of the 20μL sample volume was followed by chloroform-methanol/liquid/liquid extraction protocol (Folch extraction) with a mixture of in the proportion 2:1 (v / v) [11].

The biologically abundant compounds of (lyso-) glycerophospholipids, that is, (lyso-) glycerophosphocholines, glyceroethanolamines, glycoserines, glyceroglycerides, and sphingolipids, i.e., sphingomyelins, ceramides, dihydroceramides, and 2-hydroxietilceramides, were quantitatively analyzed through ionospheric ionization flow injection methodology using tandem mass spectrometry (FIA-ESI-MS/MS). The detection of multiple reaction monitoring (MRM) in positive and negative form was carried out using SCIEX 4000 QTRAP® equipment (SCIEX, Darmstadt, Germany). The lipid records described represent the sum of the sign of all the isobaric lipids with the same molecular weight (interval of ± 0.5 Da) within the same lipid class. Five internal standards were used to compensate for effects of the matrix and 43 external standards for the multipoint calibration. The accuracy of the measurements was in the normal interval for the methods (deviations from the target ≤ 20%) for all of the analytes. Metabolite concentrations of each sample were determined in a single analysis and concentration values are given in μM for all metabolites.

The quantitative data analysis was carried out using the MetIDQ ™ internal software, allowing for isotopic correction.

### Determining insulin resistance, metabolic syndrome, and dyslipidemia

Insulin resistance was determined using the homeostatic model for assessing insulin resistance (HOMA-IR), using the formula (HOMA-IR = insulin (mUI/L) x glycaemia (mmol/L) ÷ 22.5).Insulin resistance was determined when the HOMA-IR>2.7 [12,13].

Participants with metabolic syndrome (MS) were defined as presenting at least three out of five of these criteria: waist circumference ≥80 cm; systolic arterial pressure ≥130 and/or diastolic arterial pressure ≥85 mmHg; fasting glucose≥100mg/dL; triglycerides ≥ 150 mg/dL and HDL < 50 mg/dL, or use of antihypertensive, hypoglycemic, or lipid-lowering drugs [14]. Dyslipidemia was determined when they presented some of the following alterations: LDL ≥ 160 mg/dl; TG ≥ 150 mg/dl; and HDL < 50 mg/dl, isolated or in association [15].

### Statistical analysis

The quantitative variables are presented as the mean and standard deviation, the t-test was used to identify the significant differences between the groups, and Pearson’s coefficient (r) was used to evaluate the correlation between the variables, with values of p<0.05 being considered statistically significant. The STATA12.0 and SAS 9.4 statistical programs were used.

To analyze the lipidomic data, the in-house *MetldQTM* software from Biocrates Life Sciences AG was used to examine the raw data and evaluate and export the data. The data were processed and statistically analyzed using R (Version 3.2.3). The raw data were cleaned in order to exclude the analytes with concentration values lacking or below the limit of detection (LOD). The data were subsequently transformed into log2.

The fold change (FC) was calculated using the ratio between the means of the metabolites from post menopause (case) and pre-menopause (control) (FC = Case-Control/Control). The false discovery rate (FDR) was calculated using Benjamini-Hoochberg test was also calculated. The differences were considered notable when the analytical variance was greater than 25% between the groups; that is, FC>1.25 or FC<0.75, so that it was considered as an altered concentration in post-menopause in relation to pre-menopause [16].

## Results

### Clinical and laboratory characteristics

The sample of this study was composed of 40 participants with an average age of 44.25 (SD 2.73) in pre-menopause and 57.7 (SD 4.65) in post-menopause. The laboratory tests for total cholesterol, HDL, LDL, VLDL, triglycerides, estradiol, FSH, progesterone, and LH, presented a difference between the groups, with higher mean values in the post-menopause group (p<0.05), as the data in Table 1 show.

**Table 1:** Demographic and clinical characteristics of the women in pre-menopause and post-menopause groups. São Luís, Maranhão, 2013.

Variables	Pre menopause		Post menopause		T test
	Mean	SD	Mean	SD	
Age (year)	44.25	2.73	57.7	4.65	<0.0001*
Fasting glucose (mg/dL)	95.7	9.52	106.3	32.05	0.17
Insulin (mUI/dL)	8.96	5.15	10.16	4.25	0.43
HB glycated (%)	5.78	0.35	6.66	1.62	0.027*
Total cholesterol (mg/dL)	200.15	26.38	242.25	66.97	0.015*
HDL (mg/dL)	58.5	10.72	55.65	14.9	0.492
LDL (mg/dL)	121.45	21.49	156.85	59.45	0.02*
VLDL (mg/dL)	20.16	9.13	29.75	16.29	0.029*
Triglycerides (mg/dL)	100.95	45.62	148.75	81.27	0.029*
Estradiol (pg/dL)	191.01	84.15	5.26	0.88	<0.0001*
FSH (mUI/dL)	4.4	2.28	74.44	22.62	<0.0001*
Progesterone (ng/dL)	7.7	6.35	0.5	1.38	<0.0001*
LH (mUI/dL)	12.09	30.06	37.99	14.09	0.002*
hs-CRP (mg/L)	2.8	2.08	2.4	1.76	0.516

HB glycated = glycated hemoglobin, HDL = High density lipoprotein, LDL = Low-density lipoprotein, VLDL = Very low-density lipoprotein, FSH = follicle stimulating hormone LH = luteinizing hormone, hs-CRP = high sensitivity C-reactive protein.SD = standard deviation. T-test p<0.05*.

In the sample studied, 32.5% of the participants presented metabolic syndrome and insulin resistance, while 35% of them were diagnosed with dyslipidemia.

### Lipidomic characterization according to the menopause phases

One hundred twenty-three (123) metabolites were detected, of which 121 increased and 2 decreased in the post-menopause group compared to the pre-menopause group. However, it was verified that 40 metabolites had highly increased concentrations, a difference of over 25%, specifically ten phosphatidycholines (PC), fifteen phosphatidylethanolamines (PE), and fifteen ceramides (CER).

The fold change analysis revealed changes in the post-menopause compared to the pre-menopause group in one metabolite of (Lyso) PE and five CER, with all presenting FC>1.25. The metabolites N.C23:0.Cer, N.C23:0(OH).Cer, and N.C24:0(OH). Cer had FC values of 1.68, 1.59, and 1.58, respectively. In Table 2,the lipid species with fold change >1.25 are listed.

**Table 2:** Fold change values, referring to the difference between the concentrations of lipids of the women, detected in the post-menopause and pre-menopause groups. São Luís, Maranhão, 2013.

Classes	Metabolites	FC	p value
**Phosphatidylcholines**	LPC.a.C18:0	1.32	0.0913
	PC.aa.C36:0	1.29	0.1205
	PC.aa.C36:1	1.27	0.0932
	PC.aa.C36:5	1.3	0.5034
	PC.aa.C40:5	1.26	0.3309
	PC.ae.C36:1	1.33	0.0719
	PC.ae.C38:1	1.33	0.0648
	PC.ae.C38:2	1.31	0.0516
	PC.ae.C38:3	1.29	0.1304
	PC.ae.C40:5	1.46	0.2956
**Phosphatidylethanolamines**	LPE.a.C16:0	1.33	0.0962
	**LPE.a.C18:0**	**1.47**	0.0411*
	PE.aa.C34:1	1.35	0.1704
	PE.aa.C34:2	1.37	0.1069
	PE.aa.C36:1	1.44	0.6949
	PE.aa.C36:2	1.42	0.0549
	PE.aa.C36:3	1.38	0.0580
	PE.aa.C36:4	1.26	0.3170
	PE.aa.C36:5	1.49	0.7434
	PE.aa.C38:3	1.27	0.4890
	PE.aa.C38:5	1.37	0.1435
	PE.aa.C38:6	1.36	0.2701
	PE.aa.C40:5	1.46	0.6327
	PE.aa.C40:6	1.45	0.1933
	PE.ae.C36:1	1.28	0.1262
**Ceramides**	**N.C10:0.Cer**	**1.68**	0.0314*
	N.C12:0.Cer	1.28	0.2679
	N.C18:0(OH).Cer.2H.	1.28	0.7598
	N.C20:0.(OH).Cer	1.47	0.7362
	N.C21:0.Cer	1.54	0.0731
	N.C22:0(OH).Cer	1.47	0.2969
	N.C22:0(OH).Cer.2H.	1.34	0.9543
	**N.C23:0.Cer**	**1.34**	0.0421*
	N.C23:0.Cer.2H.	1.28	0.3009
	**N.C23:0.(OH).Cer**	**1.59**	0.0194*
	**N.C24:0.(OH).Cer**	**1.58**	0.0146*
	N.C24:0(OH).Cer.2H.	1.37	0.4110
	**N.C25:0.Cer**	**1.35**	0.0478*
	N.C25:0.Cer.2H.	1.39	0.6782
	N.C25:1.Cer	1.49	0.1232

FC = *Fold change*

*p<0.05.; FDR = 0.28.

Of the forty lipid metabolites, fourteen presented a significant difference in the mean concentrations (p <0.05) between the groups, as presented in Table 3. More data are available in S1 Table.

**Table 3:** Average concentrations (μM) of lipid^a^ species among in the women, in the pre-menopause and post-menopause. São Luís, Maranhão, 2013.

Variables		Pre Menopause			Post Menopause			
		Mean	SD	Variation	Mean	SD	Variation	T test
Phosphatidylcholines Phosphatidylethanolamines	PC.ae.C36:1	7.25	(2.65)	0.37–12.87	9.67	(4.24)	5.23–21.33	0.0374*
	PC.ae.C38:1	5.54	(1.75)	0.44–8.28	7.34	(3.14)	4.11–14.22	0.0311*
	PC.ae.C38:2	5.27	(1.58)	0.57–7.40	6.92	(2.59)	3.83–11.91	0.0206*
	LPE.a.C18:0	0.65	(0.21)	0.33–1.03	0.96	(0.59)	0.34–2.93	0.0337*
	PE.aa.C36:1	1.02	(0.48)	0.33–1.87	1.47	(0.83)	0.47–3.24	0.0423*
	PE.aa.C36:2	3.27	(1.65)	0.92–7.35	4.67	(2.58)	1.88–11.41	0.0498*
	PE.aa.C36:3	1.57	(0.66)	0.54–2.98	2.17	(1.06)	0.66–4.57	0.0380*
Ceramides	N.C10:0.Cer	0.12	(0.04)	0.05–0.22	0.21	(0.17)	0.07–0.85	0.0444*
	N.C22:0(OH).Cer	0.01	(0.01)	0.001–0.22	0.02	(0.01)	0.001–0.05	0.0461*
	N.C23:0.Cer	0.76	(0.31)	0.27–1.57	1.02	(0.43)	0.42–1.89	0.0364*
	N.C23:0(OH).Cer	0.02	(0.01)	0.01–0.04	0.03	(0.02)	0.01–0.08	0.0149*
	N.C24:0(OH).Cer	0.05	(0.04)	0.01–0.09	0.07	(0.04)	0.02–0.18	0.0207*
	N.C25:0.Cer	0.21	(0.08)	0.07–0.38	0.29	(0.13)	0.14–0.58	0.0417*
	N.C25:1.Cer	0.02	(0.01)	0.001–0.04	0.03	(0.02)	0.001–0.08	0.0400*

^a^With statistical difference between groups. T-test

*p<0.05; SD = standard deviation; Variation (min-max).

Among the circulating CER that presented higher concentrations in the serum were the species N.C23:0.Cer, N.C25:0-Cer, and N.C10.0:Cer, in addition to the non-ceramides PC.ae.C36:1 and PE.aa.C36:2.

### Correlation between lipidomics and glycidic and lipid markers

The relationship between lipidomics and different biochemical parameters was assessed with the aim of investigating whether specific alterations in lipids are associated with these markers. The data from this analysis are summarized in S2 Table. This evaluation revealed positive correlations with fasting glucose, glycated hemoglobin, total cholesterol, LDL cholesterol, non-HDL cholesterol, and triglycerides, the majority of the associations being with ceramides.

Strong correlations were also observed between PC.ae.C38:1 and PC.ae.C38:2 and total cholesterol (*r = 0*.*65 and r = 0*.*60 p<0*.*001*, respectively). Moreover, triglyceride levels were correlated with both PE.aa.C36:1 and PE.aa.C36:2(*r = 0*.*75 and r = 0*.*67*, *p<0*.*001*, respectively).LPE.a.C18:0was positively associated with fasting glucose (*r = 0*.*73 p<0*.*001*) and glycated hemoglobin (*r = 0*.*75 p<0*.*001*).

In the case of the ceramides, N.C10:0.Cer was associated with fasting glucose(*r = 0*.*83*, *p<0*.*001*) and glycated hemoglobin (*r = 0*.*81*, *p<0*.*001)*. The other species correlated with total cholesterol, LDL, and triglycerides.

## Discussion

This study investigated the influence of phenotypic factors in the composition of lipids in the serum of pre-menopausal and post-menopausal women. The lipid profile evaluated by the current classic methods, represented by total cholesterol, LDL, VLDL, and triglycerides, was changed with a significant difference between the menopausal phases; these alterations can be explained by the decrease in estradiol, progesterone, and by being overweight and obese in post-menopause [17], and the results could also be due to weight gain and insulin resistance [1].

It was observed that in the post-menopause group, there was also an increase in the concentration of glycated hemoglobin; however, aging in itself is associated with an increased risk of type II diabetes mellitus (DM2), as well as obesity, sedentarism, and smoking, among others [18]. In the sample studied, obesity was also observed, helping to explain the increase in glycated hemoglobin.

The studies Heart and Estrogen/progestin Replacement Study and Women’s Health Initiative showed a reduction in the incidence of DM2 during hormone replacement therapy, suggesting an influence of the hormones on glycidic metabolism [19,20].

The importance of lipids such as triglycerides and cholesterol as a marker for predicting cardiovascular disease is already well known, but modern lipid analysis shows that the lipidomic profile of human plasma includes a variety of individual species [4]; however, these species have still not been well explored, due to technical limitations such as physical-chemical differences in lipid species, which make their simultaneous characterization using analytical techniques difficult [21].

By evaluating the concentrations of lipid metabolites identified in the lipidomic analysis, differences between the phases of menopause were observed, with 14 lipids presenting a significant increase in the post-menopause group, where the ceramide class was in greater number but lower concentration, since sphingolipids present a very low abundance in plasma [22].

Ceramides are precursors of the complex sphingolipids, and various studies suggest that they are an important factor to insulin resistance, cardiovascular disease and other morbidities that occur in women, especially in post-menopausal women[22–27].

In the study carried out by Ke et al. [2] with climacteric women, lipids were identified as potential biomarkers, with the species phosphatidylcholine (LPC), lysophosphatidylethanolamine (LPE), fatty acids, and acylcarnitines standing out in higher concentrations in post-menopausal women. In our study, we also identified metabolites of these classes; however, the species of LPC and LPE differed from those that we identified as changed in post-menopausal women, possibly due to the lipid extraction methods (acetonitrile x MeOH / CHCl3) [2], the technology used in the detection (UPLC-QTOF/MS x FIA-ESI-MS/MS), as well as the biological sources of the population studied.

Wallace and colleagues [28] evaluated the relationship among lipidomic, inflammatory markers, and insulin resistance in people of both genders and found that CER, PC, and PE are different in women. In our study, an increase in the same classes was also identified in the post-menopause group.

Alterations were identified in 5 species of CER in the post-menopausal group. These findings might be an indication that some of these women may be at risk of developing a cardiovascular disease, because, as the study published by Laaksonen et al. [29] shows, CER are significantly associated with death by CVD, suggesting that the detection of CER in plasma is a better marker than those currently used. Thus, this finding important for identifying patients at high risk of CVD and who need immediate and effective therapeutic interventions.

Previous studies suggest that CER increase with age and are associated with accelerated aging and chronic diseases related to age, particularly cardiovascular and metabolic diseases [9, 16, 21].

Our results are in agreement with various studies already carried out in animal [30] and human models, which have shown that the plasmatic levels of ceramides are correlated with aerobic capacity [31], cardio-respiratory capacity [26], diabetes, and pre-diabetes[16, 22], showing a relationship with glycidic and lipid profiles.

Differences were also detected in the PC and PE lipids between the groups, which constitute the most abundant phospholipids in mammals and are also indicated as probable modulators of muscular insulin resistance [22]. The most significant alterations in the PPC class were those that had ether links, as already reported by Weir et al. [32],suggesting that ether phospholipids are linked with obese individuals, which may explain our findings, although these lipids do not present any correlation with BMI.

Plasmatic lipidomic studies in human beings have also shown a clear association between PE and obesity[32], pre-diabetes, and type 2 diabetes mellitus [33], as well as an association between a low proportion of PC/PE in the liver and non-alcoholic fatty hepatic disease [22].

Important associations were observed between the lipids and laboratory dosages of metabolic markers in the sample studied, including the correlations between metabolites, namely, LPE.a.C18:0 and N.C10:0.Cerand fasting glucose and glycated hemoglobin.

These associations for species of LPE and ceramides were reported by Meikle et al.[33], in the study *Australian diabetes*, *obesity and lifestyle*, where these classes were significantly associated with the presence of type 2 diabetes (DT2) and pre-diabetes and observed by Tulipani et al. [16] in a study with insulin-resistant participants in the pre-diabetic stage, which confirmed correlations with circulating sphingolipids, including various specific ceramides. These findings implicate the ceramide biosynthesis pathway as an important metabolic process in DT2 and insulin resistance. The results led the authors to believe that lipidomic plasmatic analysis may have an independent and additional use in detecting insulin resistance and central obesity [16, 33].

Other CER correlated with total cholesterol, LDL, and triglycerides, which has been observed in previous studies [26, 27].

The clinical relevance of our results, despite being preliminary, are original and enable us to speculate possibilities for detecting markers that can identify the metabolic alterations that post-menopausal women undergo at an earlier stage.

Thus, these data can be considered relevant because they suggest the importance of alterations in lipid classes, especially ceramides, which have already been indicated by various authors as possible target lipids for preventing and treating CVD in post-menopausal women, who are at high risk for these diseases. However, more research will be needed to corroborate these results.

Some limitations of our study should be addressed, including the relatively small sample, failure to perform analyses of other lipid categories and possible lipid changes observed due to other reasons, including biological sources and uncontrolled confounding factors.

## Conclusion

In this study, differences were observed in the concentrations of lipid species, especially ceramides, between pre- and post-menopausal women, as well as correlations with classic glycidic and lipid markers, which may in the future be useful to investigate diseases associated with menopause.

## Supporting information

S1 Table Concentration (μM) of all the lipid species identified whose FC> 1.25 among the groups of women, in the post-menopause and pre-menopause groups. São Luís, 2013.
(DOCX)

S2 Table Correlation of the biochemical markers with lipid species in the plasma of the women participating in the study. São Luís, 2013.
(DOCX)
