# Supplementary material for: Alterations in postmenopausal plasmatic lipidome
Source: PLoS One. 2018 Sep 4;13(9):e0203027. doi: 10.1371/journal.pone.0203027 (PMC6122933; doi:10.1371/journal.pone.0203027)
Supplement: S1 Table — São Luís, 2013. (DOCX) [file pone.0203027.s001.docx]

Table S1 - Concentration (µM) of all the lipid species identified whose FC> 1.25 among the groups of women, in the post-menopause and pre-menopause groups. São Luís, 2013.

| **Variables** | | **Pre Menopause** | | |  | **Post Menopause** | | | **T test** |
| --- | --- | --- | --- | --- | --- | --- | --- | --- | --- |
|  |  | **Mean** | **SD** | **Variation** |  | **Mean** | **SD** | **Variation** |  |
| **Phosphatidylcholines** | LPC.a.C18:0 | 63.42 | (20.27) | 31.87-100.68 |  | 83.79 | (40.18) | 31.77-177.06 | 0.0501 |
|  | PC.aa.C36:0 | 7.45 | (2.37) | 3.54-12.13 |  | 9.61 | (4.82) | 4.77-20.41 | 0.0801 |
|  | PC.aa.C36:1 | 45.33 | (14.83) | 17.28-73.2 |  | 57.67 | (26.76) | 31.18-131.77 | 0.0792 |
|  | PC.aa.C36:5 | 21.51 | (11.88) | 8.02-51.87 |  | 27.87 | (22.03) | 6.74-94.38 | 0.2628 |
|  | PC.aa.C40:5 | 22.19 | (8.12) | 11.81-38.93 |  | 27.87 | (17.11) | 11.15-68.59 | 0.1881 |
|  | PC.ae.C36:1 | 7.25 | (2.65) | 0.37-12.87 |  | 9.67 | (4.24) | 5.23-21.33 | 0.0374* |
|  | PC.ae.C38:1 | 5.54 | (1.75) | 0.44-8.28 |  | 7.34 | (3.14) | 4.11-14.22 | 0.0311* |
|  | PC.ae.C38:2 | 5.27 | (1.58) | 0.57-7.40 |  | 6.92 | (2.59) | 3.83-11.91 | 0.0206* |
|  | PC.ae.C38:3 | 4.82 | (1.56) | 0.34-7.11 |  | 6.22 | (3.04) | 2.97-14.78 | 0.0757 |
|  | PC.ae.C40:5 | 4.60 | (1.60) | 0.12-7.13 |  | 6.71 | (5.63) | 0.49-25.91 | 0.1143 |
| **Phosphatidylethanolamines** | LPE.a.C16:0 | 0.32 | (0.10) | 0.16-0.51 |  | 0.42 | (0.24) | 0.19-1.22 | 0.0842 |
|  | LPE.a.C18:0 | 0.65 | (0.21) | 0.33-1.03 |  | 0.96 | (0.59) | 0.34-2.93 | 0.0337* |
|  | PE.aa.C34:1 | 0.89 | (0.53) | 0.23-1.97 |  | 1.21 | (0.81) | 0.36-3.09 | 0.1592 |
|  | PE.aa.C34:2 | 1.71 | (0.96) | 0.48-4.17 |  | 2.35 | (1.42) | 0.83-6.13 | 0.1057 |
|  | PE.aa.C36:1 | 1.02 | (0.48) | 0.33-1.87 |  | 1.47 | (0.83) | 0.47-3.24 | 0.0423* |
|  | PE.aa.C36:2 | 3.27 | (1.65) | 0.92-7.35 |  | 4.67 | (2.58) | 1.88-11.41 | 0.0498* |
|  | PE.aa.C36:3 | 1.57 | (0.66) | 0.54-2.98 |  | 2.17 | (1.06) | 0.66-4.57 | 0.0380* |
|  | PE.aa.C36:4 | 3.06 | (1.26) | 1.35-5.82 |  | 3.85 | (2.34) | 1.13-9.69 | 0.1880 |
|  | PE.aa.C36:5 | 0.45 | (0.24) | 0.02-0.86 |  | 0.67 | (0.54) | 0.003-2.06 | 0.1063 |
|  | PE.aa.C38:3 | 2.42 | (1.09) | 0.14-4.21 |  | 3.06 | (1.97) | 0.19-8.66 | 0.2044 |
|  | PE.aa.C38:5 | 3.67 | (1.37) | 1.82-6.83 |  | 5.03 | (3.05) | 1.59-12.74 | 0.0767 |
|  | PE.aa.C38:6 | 6.94 | (2.80) | 2.88-13.33 |  | 9.43 | (7.13) | 2.74-31.32 | 0.1555 |
|  | PE.aa.C40:5 | 1.86 | (1.05) | 0.05-3.63 |  | 2.71 | (2.17) | 0.007-8.64 | 0.1224 |
|  | PE.aa.C40:6 | 4.35 | (1.77) | 1.99-7.34 |  | 6.30 | (5.10) | 1.83-22.1 | 0.1152 |
|  | PE.ae.C36:1 | 0.14 | (0.08) | 0.002-0.28 |  | 0.18 | (0.08) | 0.02-0.32 | 0.1297 |
| **Ceramides** | N.C10:0.Cer | 0.12 | (0.04) | 0.05-0.22 |  | 0.21 | (0.17) | 0.07-0.85 | 0.0444* |
|  | N.C12:0.Cer | 0.67 | (0.20) | 0.36-1.02 |  | 0.86 | (0.57) | 0.34-2.77 | 0.1675 |
|  | N.C18:0(OH).Cer.2H. | 0.01 | (0.0006) | 0.0001 -0.003 |  | 0.002 | (0.001) | 0.001-0.005 | 0.2070 |
|  | N.C20:0(OH).Cer | 0.03 | (0.01) | 0.012-0.061 |  | 0.04 | (0.06) | 0.0004-0.28 | 0.2767 |
|  | N.C21:0.Cer | 0.02 | (0.01) | 0.0001-0.037 |  | 0.014 | (0.01) | 0.001-0.05 | 0.0553 |
|  | N.C22:0(OH).Cer | 0.01 | (0.01) | 0.001-0.22 |  | 0.02 | (0.01) | 0.001-0.05 | 0.0461* |
|  | N.C22:0(OH).Cer.2H. | 0.0008 | (0.0006) | 0.0002-0.0021 |  | 0.0011 | (0.001) | 0.00001-0.004 | 0.3755 |
|  | N.C23:0.Cer | 0.76 | (0.31) | 0.27-1.57 |  | 1.02 | (0.43) | 0.42-1.89 | 0.0364* |
|  | N.C23:0.Cer.2H. | 0.07 | (0.03) | 0.02-0.15 |  | 0.09 | (0.06) | 0.03-0.22 | 0.1798 |
|  | N.C23:0(OH).Cer | 0.02 | (0.01) | 0.01-0.04 |  | 0.03 | (0.02) | 0.01-0.08 | 0.0149* |
|  | N.C24:0(OH).Cer | 0.05 | (0.04) | 0.01-0.09 |  | 0.07 | (0.04) | 0.02-0.18 | 0.0207* |
|  | N.C24:0(OH).Cer.2H. | 0.001 | (0.001) | 0.00008-0.0037 |  | 0.002 | (0.002) | 0.0001-0.007 | 0.1596 |
|  | N.C25:0.Cer | 0.21 | (0.08) | 0.07-0.38 |  | 0.29 | (0.13) | 0.14 -0.58 | 0.0417* |
|  | N.C25:0.Cer.2H. | 0.015 | (0.011) | 0.0007-0.037 |  | 0.021 | 0.017 | 0.0001-0.05 | 0.1936 |
|  | N.C25:1.Cer | 0.02 | (0.01) | 0.001-0.04 |  | 0.03 | (0.02) | 0.001-0.08 | 0.0400* |

T test, *p<0,05; SD= standard deviation; Variation (min-max).
