# Supplementary material for: Alterations in postmenopausal plasmatic lipidome
Source: PLoS One. 2018 Sep 4;13(9):e0203027. doi: 10.1371/journal.pone.0203027 (PMC6122933; doi:10.1371/journal.pone.0203027)
Supplement: S2 Table — São Luís, 2013. (DOCX) [file pone.0203027.s002.docx]

Table S2 - Correlation of the biochemical markers with lipid species in the plasma of the women participating in the study. São Luís, 2013.

| Variables | Fasting glucose | | Glycated hemoglobin | | Total cholesterol | | LDL | | HDL | | Triglycerides | |  |
| --- | --- | --- | --- | --- | --- | --- | --- | --- | --- | --- | --- | --- | --- |
|  | r | p value | r | p value | r | p value | r | p value | r | p value | r | p value | |
| LPE a C18:0 | 0.73 | 0.0000 | 0.75 | 0.0000 | 0.31 | ns | 0.25 | ns | -0.08 | ns | 0.44 | 0.0045 | |
| PC.ae.C36:1 | 0.56 | 0.0002 | 0.54 | 0.0003 | 0.56 | 0.0002 | 0.51 | 0.0006 | -0.12 | ns | 0.50 | 0.0008 | |
| PC ae C38:1 | 0.42 | 0.0061 | 0.41 | 0.0079 | 0.65 | 0.0000 | 0.64 | 0.0000 | -0.19 | ns | 0.51 | 0.0008 | |
| PC ae C38:2 | 0.40 | 0.009 | 0.40 | 0.0114 | 0.6 | 0.0000 | 0.59 | 0.0000 | -0.14 | ns | 0.44 | 0.0037 | |
| PE aa C36:1 | 0.43 | 0.0054 | 0.50 | 0.001 | 0.58 | 0.0001 | 0.42 | 0.0062 | 0.10 | ns | 0.75 | 0.0000 | |
| PE aa C36:2 | 0.38 | 0.0141 | 0.46 | 0.0024 | 0.54 | 0.0003 | 0.40 | 0.0096 | 0.08 | ns | 0.67 | 0.0000 | |
| PE.aa.C36:3 | 0.24 | ns | 0.37 | ns | 0.52 | 0.0004 | 0.36 | ns | 0.24 | ns | 0.59 | 0.0001 | |
| N-C10:0-Cer | 0.83 | 0.0000 | 0.81 | 0.0000 | 0.3 | ns | 0.25 | ns | -0.15 | ns | 0.44 | 0.004 | |
| N-C22:0(OH)-Cer | 0.22 | ns | 0.25 | ns | 0.65 | 0.0000 | 0.63 | 0.0000 | -0.18 | ns | 0.55 | 0.004 | |
| N-C23:0(OH)-Cer | 0.20 | ns | 0.18 | ns | 0.71 | 0.0000 | 0.70 | 0.0000 | -0.26 | ns | 0.62 | 0.0000 | |
| N-C23:0-Cer | 0.44 | 0.0039 | 0.41 | 0.0081 | 0.66 | 0.0000 | 0.61 | 0.0000 | -0.17 | ns | 0.66 | 0.0000 | |
| N-C24:0(OH)-Cer | 0.23 | ns | 0.24 | ns | 0.76 | 0.0000 | 0.75 | 0.0000 | -0.25 | ns | 0.62 | 0.0000 | |
| N-C25:0-Cer | 0.24 | ns | 0.22 | ns | 0.69 | 0.0000 | 0.69 | 0.0000 | -0.26 | ns | 0.57 | 0.0001 | |
| N-C25:1-Cer | 0.49 | 0.0013 | 0.47 | 0.0019 | 0.58 | 0.0001 | 0.56 | 0.0002 | -0.25 | ns | 0.62 | 0.0000 | |

HDL= High-density lipoprotein, LDL= Low-density lipoprotein ns= no statistically significant; *r* = Pearson’s coefficient, p<0.05*.
